# Incidence of Pneumoperitoneum After Gastrostomy Tube Removal

**DOI:** 10.7759/cureus.47684

**Published:** 2023-10-25

**Authors:** Anas Mahmoud, Nizar Alyassin, Eyad Baghal, Ruhin Yuridullah, Yana Cavanagh, Matthew A Grossman

**Affiliations:** 1 Internal Medicine, St. Joseph's Regional Medical Center, Paterson, USA; 2 Gastroenterology, St. Joseph's Regional Medical Center, Paterson, USA; 3 Interventional Gastroenterology, St. Joseph's Regional Medical Center, Paterson, USA

**Keywords:** gastrostomy tube removal, percutaneous endoscopic gastrostomy (peg), nonsurgical pneumoperitoneum, silent pneumoperitoneum, gastrostomy tube

## Abstract

We present the case of an 88-year-old man with a previous medical history of severe colitis and colonic strictures who presented with hematemesis. The patient was found to have a lower esophageal ulcer without any signs of perforation. Esophagogastroduodenoscopy (EGD) revealed a scar in the greater curvature of the stomach from a previously removed gastrostomy tube two months prior. On CT imaging, an incidental finding of pneumoperitoneum was also found alongside stomach perforation near the healing scar. Due to the lack of evidence of any other colonic perforation, the patient was believed to have developed this pneumoperitoneum status post-gastrectomy tube removal two months prior to presentation. Pneumoperitoneum has a wide range of presenting symptoms that vary in severity and nature, and our patient failed to present with any physical or laboratory signs of infection. Over the course of the next four months, the patient was monitored with serial CT scans, during which the pneumoperitoneum resolved. In this report, we present a case of a patient who was found to develop pneumoperitoneum post-gastric tube removal and its complete resolution without surgical or procedural intervention.

## Introduction

The percutaneous endoscopic gastrostomy (PEG) procedure is invasive, regardless of the method undertaken. The gastrostomy tube must be placed through several layers of skin, muscle, and gastric tissue and warrants the application of high intragastric pressure [1]. This pressure creates a risk of pneumoperitoneum during this procedure. The pneumoperitoneum is simply defined as air within the peritoneal cavity but outside the visceral organs [2]. Patients may present with positive clinical signs such as fever, abdominal tenderness, or leukocytosis [3]. Although they are infrequent, when a pneumoperitoneum occurs, it tends to be benign or self-limiting [1]. These are called spontaneous or non-surgical due to the lack of positive clinical signs [2]. When positive signs are present due to the air causing peritoneal inflammation, further workups or therapeutic measures are usually taken. At times, the pneumoperitoneum is due to perforation, but in about 5-15% of cases, it is due to another cause and does not warrant an emergent surgical correction [3]. The published work today surrounding PEG procedures and pneumoperitoneum is secondary to the insertion, and no publication today, to the best of our knowledge, has stated that pneumoperitoneum was found secondary to the removal of a gastric tube. Because of this, we present a case of spontaneous pneumoperitoneum in an 88-year-old male patient secondary to the removal of a gastric tube with a healing scar from a previous PEG procedure.

## Case presentation

**Figure 1:**
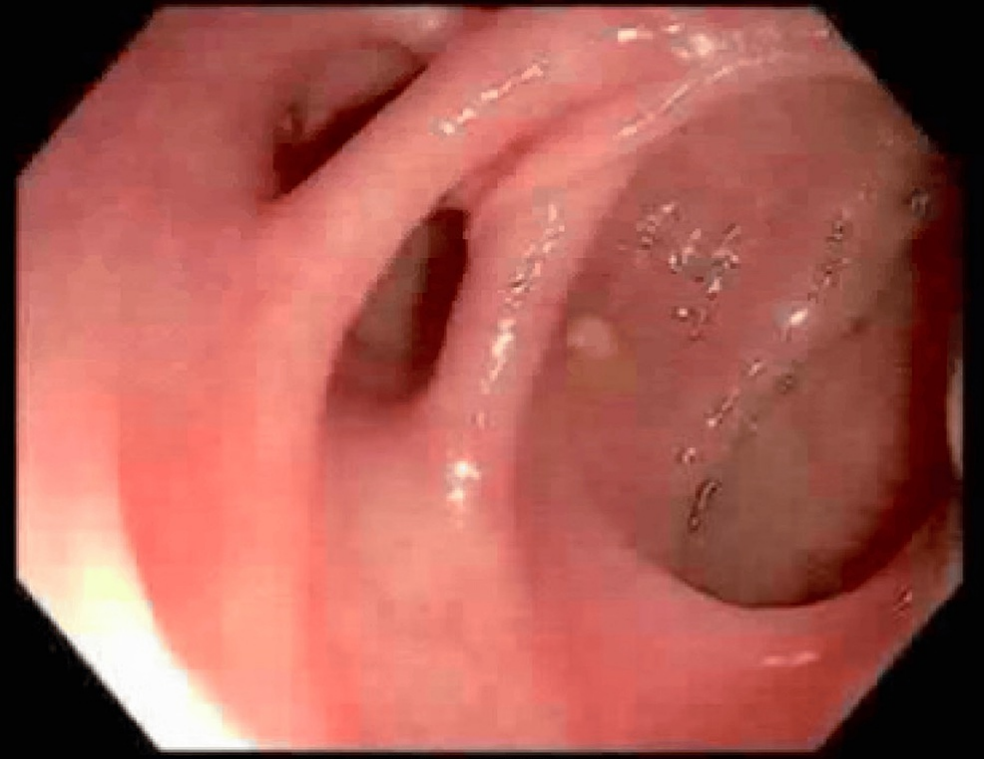
Colonoscopy of the sigmoid colon showing diverticulosis

**Figure 2:**
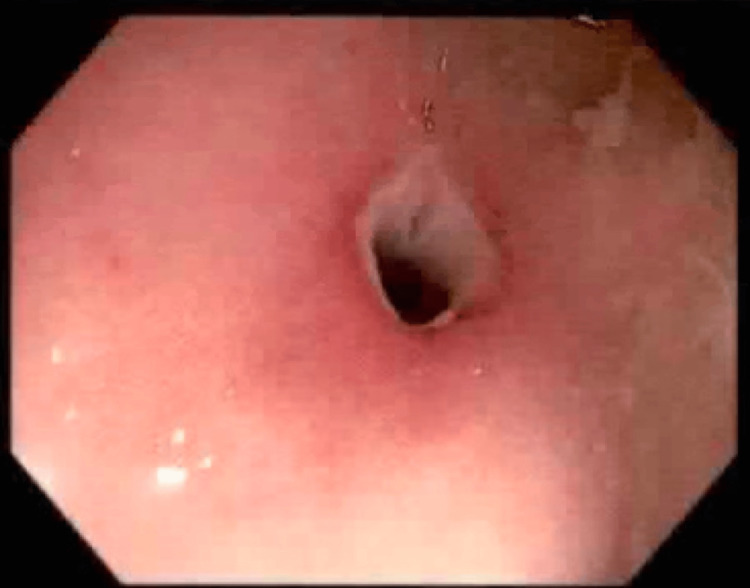
Colonoscopy - splenic flexure showing stricture

**Figure 3:**
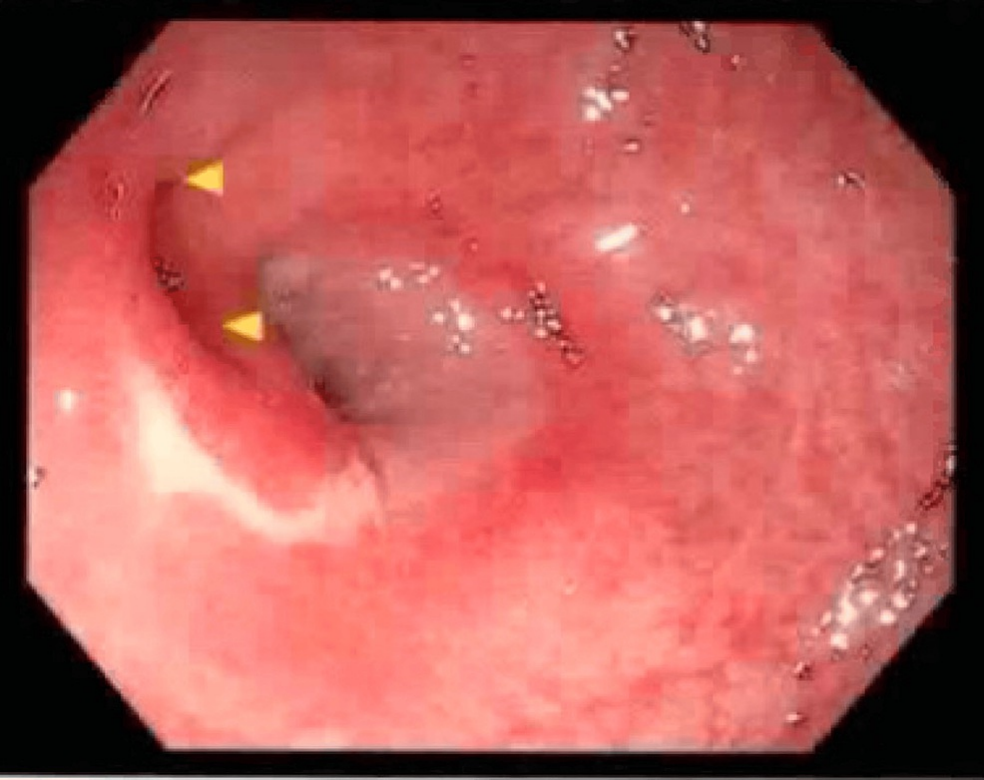
Colonoscopy - cecum stricture

**Figure 4:**
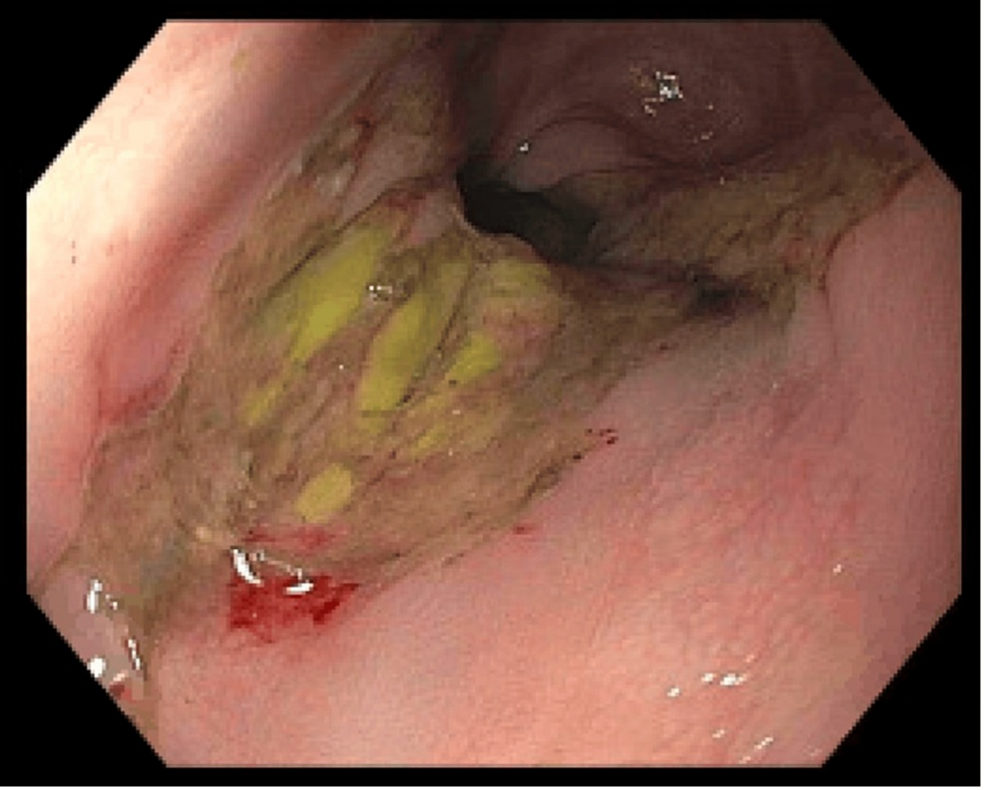
Esophagogastroduodenoscopy - esophageal ulcer with no stigmata of recent bleeding and grade C reflux esophagitis

**Figure 5:**
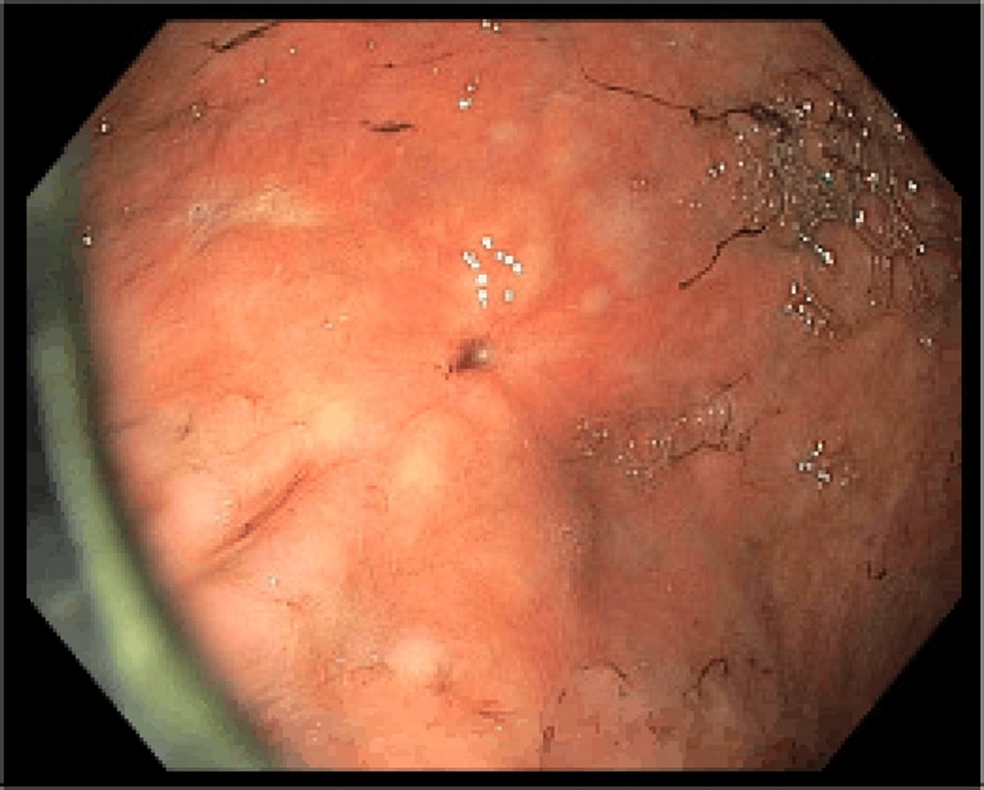
Esophagogastroduodenoscopy - a 5 mm previous percutaneous endoscopic gastrostomy tube scar on the greater curvature of the stomach

**Figure 6:**
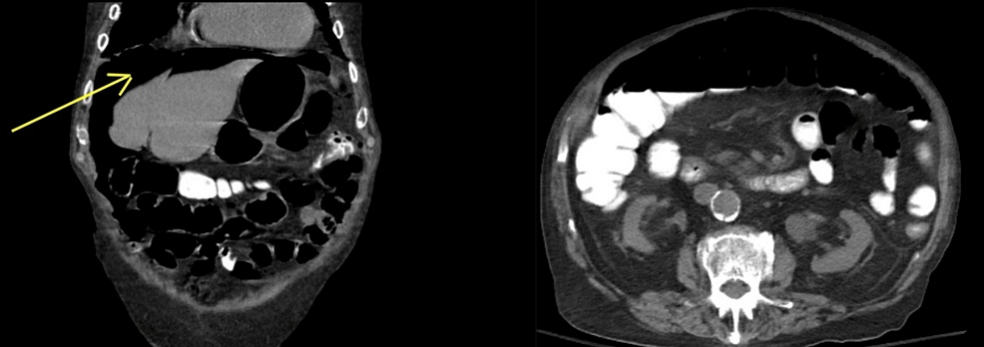
CT abdomen/pelvis showing large pneumoperitoneum

**Figure 7:**
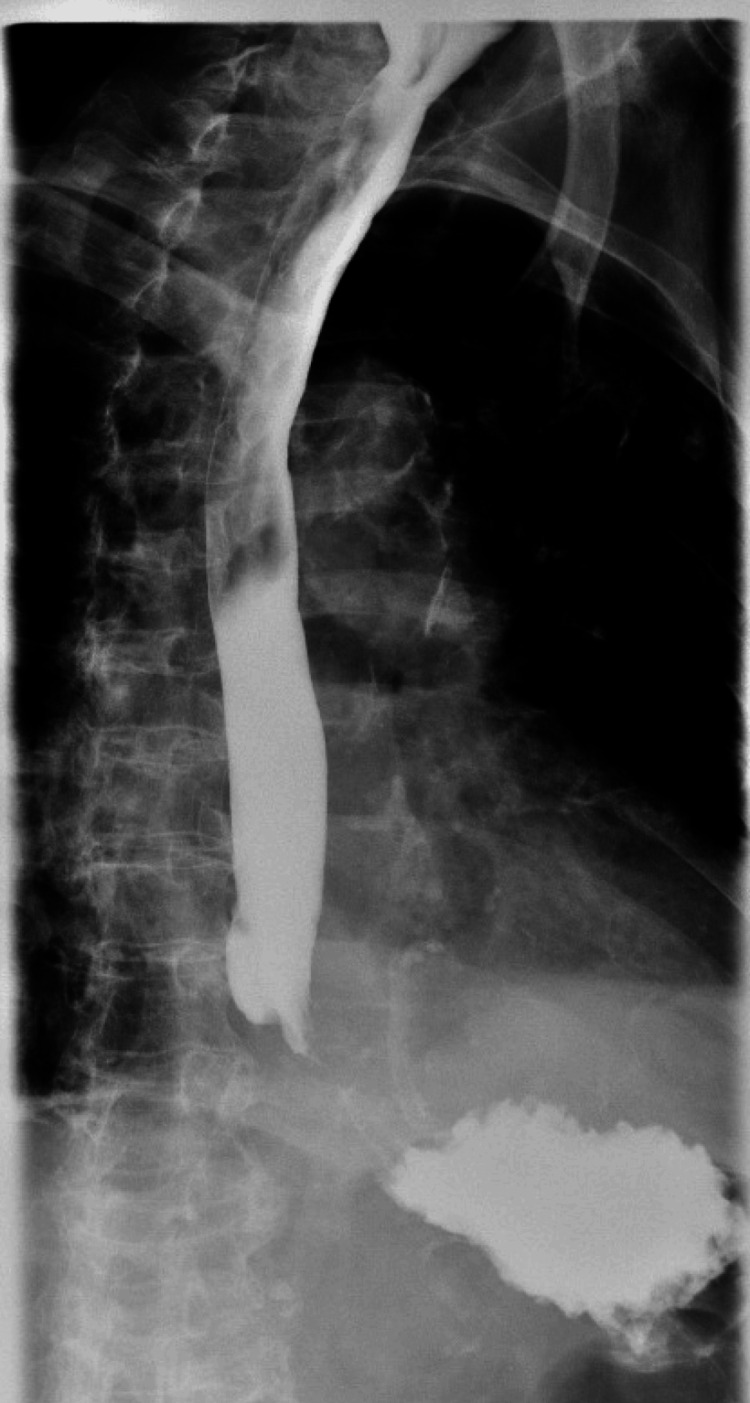
Kidneys, ureters, bladder with upper gastrointestinal series showing no obstruction or leak

**Figure 8:**
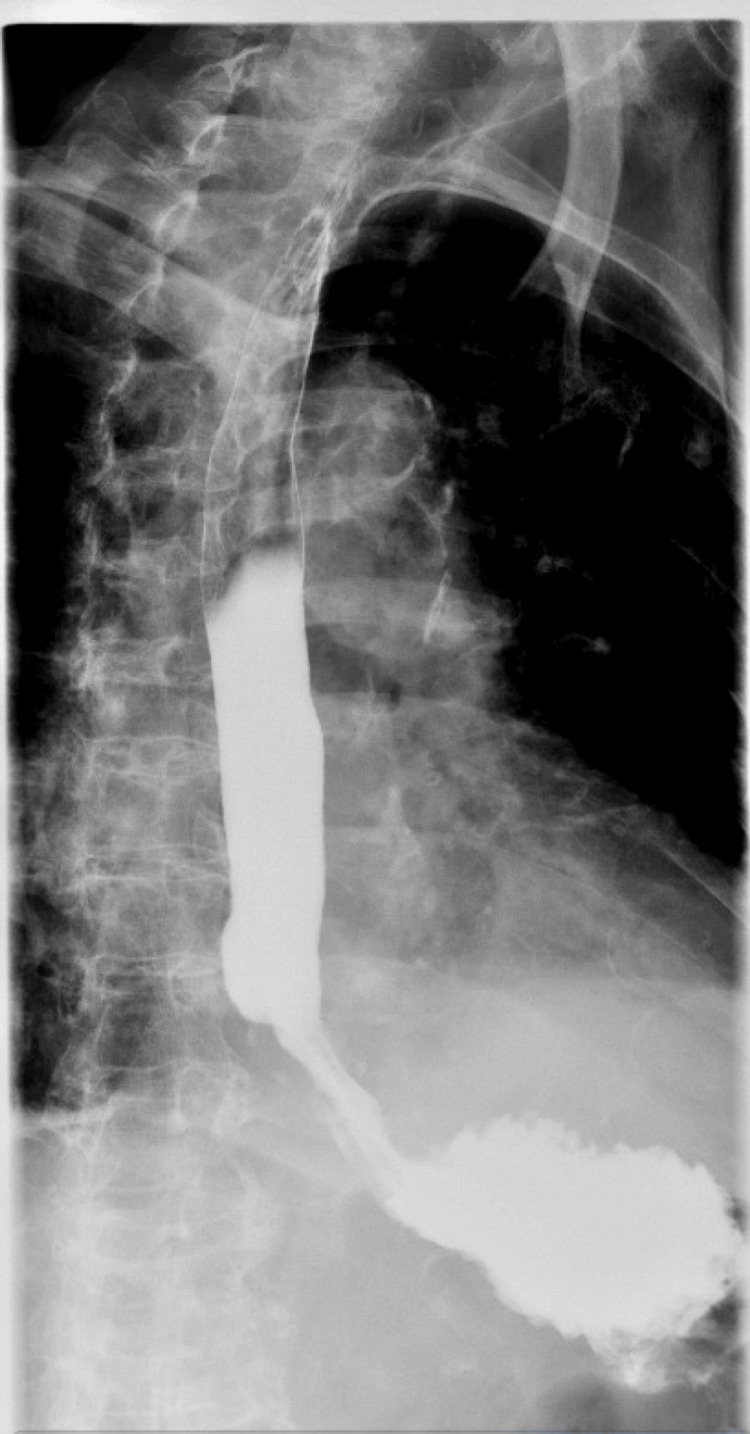
Kidneys, ureters, bladder with upper gastrointestinal series showing no obstruction or leak

**Figure 9:**
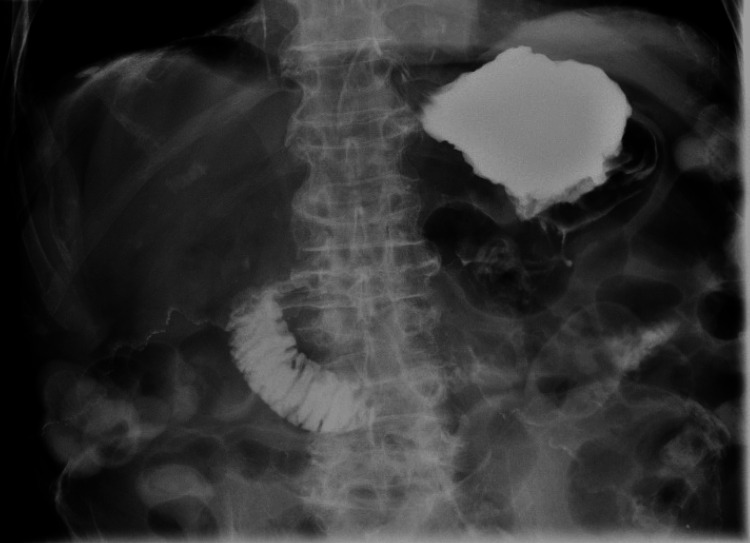
Kidneys, ureters, bladder with upper gastrointestinal series showing no obstruction or leak

**Figure 10:**
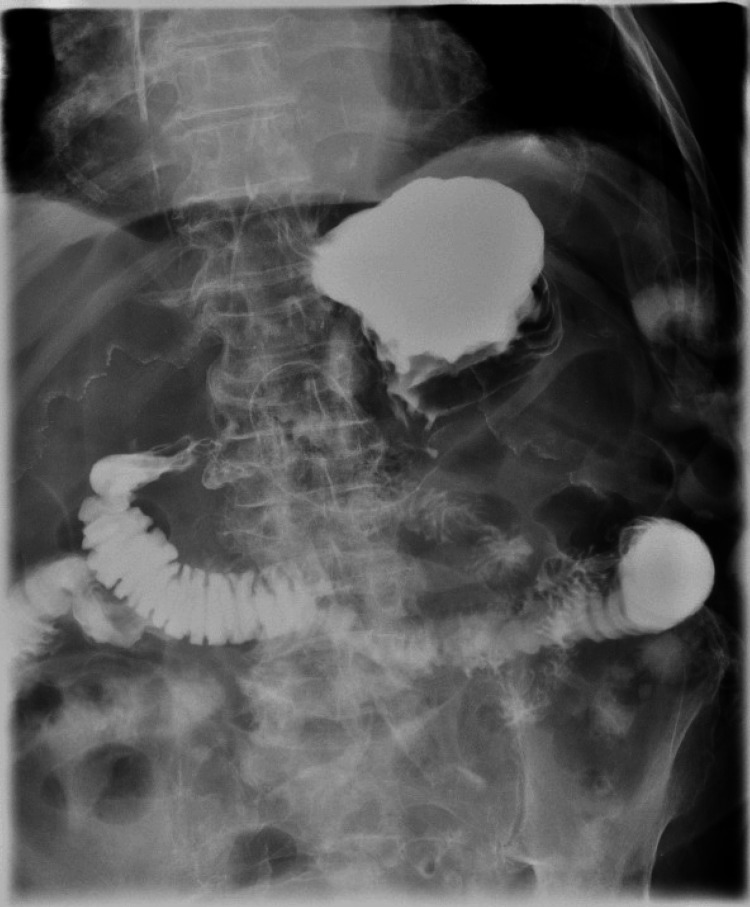
Kidneys, ureters, bladder with upper gastrointestinal series showing no obstruction or leak

**Figure 11:**
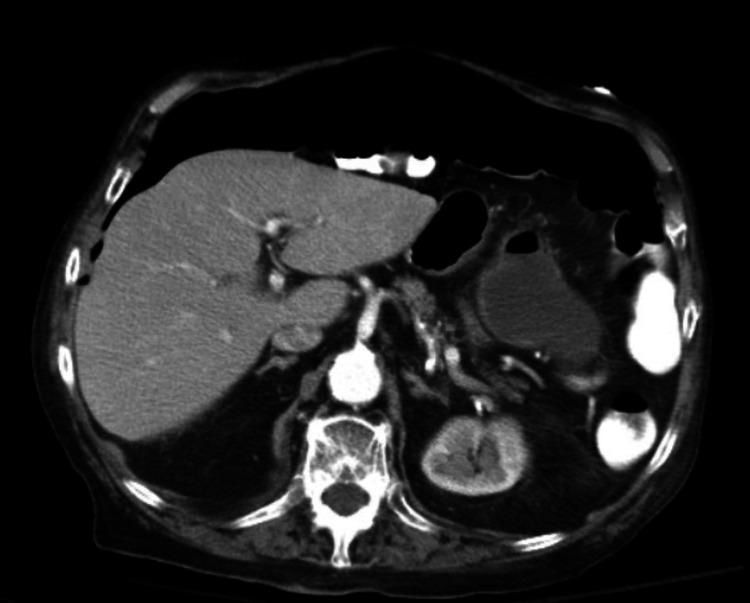
CT abdomen showing stomach having a oval 7.6 cm × 4 cm × 5 cm fluid collection with internal content debris and air bubbles along the greater curvature

**Figure 12:**
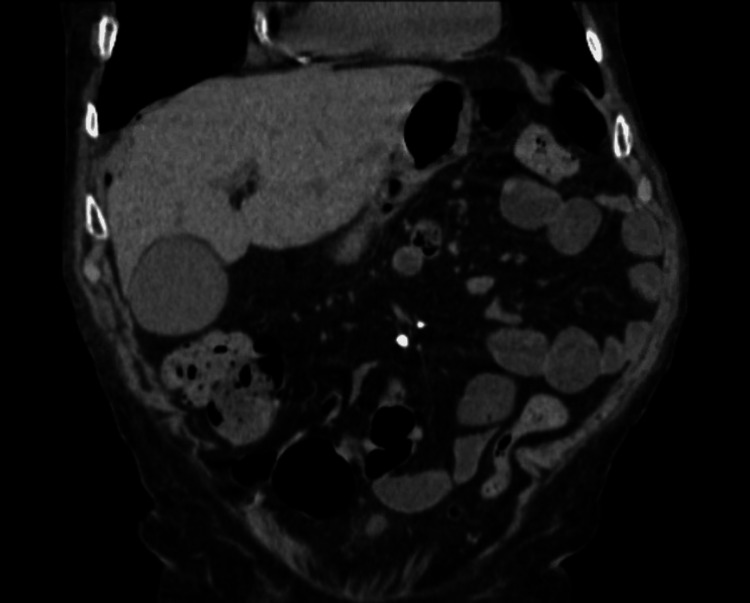
CT abdomen/pelvis showing resolving fluid collection in the stomach and decreased pneumoperitoneum

**Figure 13:**
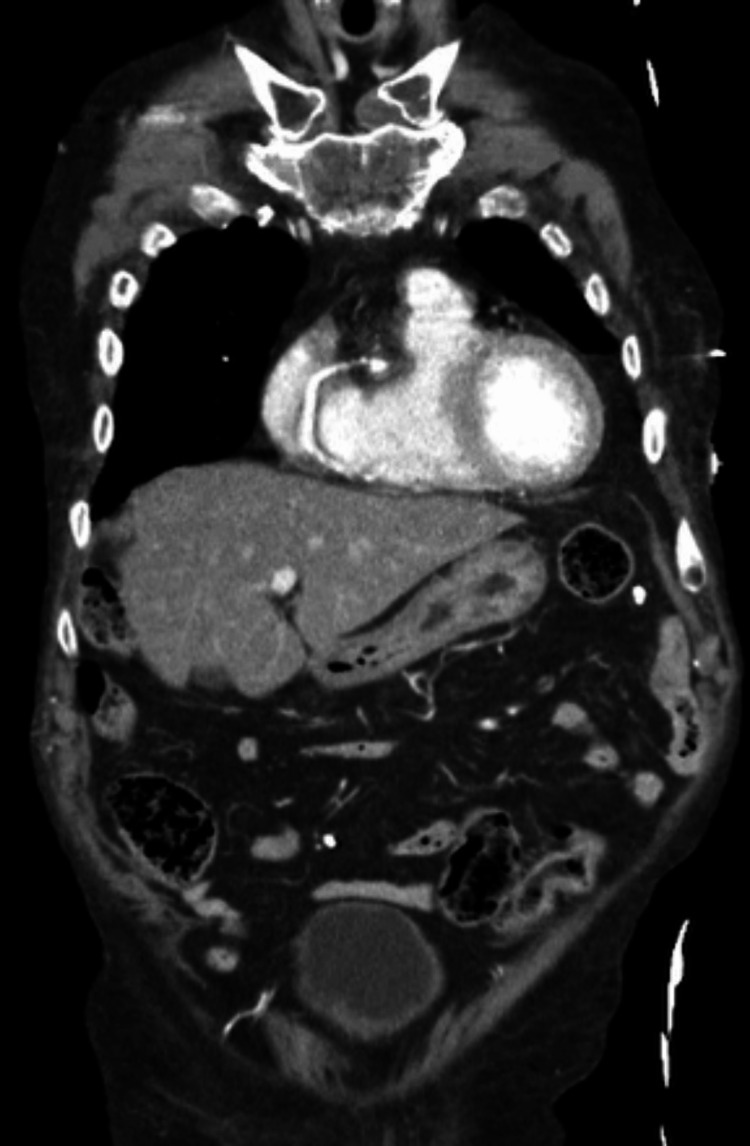
CT abdomen/pelvis showing resolved pneumoperitoneum

An 88-year-old male with a previous history of colitis and colonic strictures presented to the hospital after an episode of hematemesis. The patient had been treated for colonic strictures with balloon dilators two years prior (Figures 1-3). On presentation, he admitted to having one episode of hematemesis but denied any black, tarry stool or any signs of blood in the stool. Although his physical examination was unremarkable and he had normal blood pressure and heart rate, his hemoglobin was found to be 6 g/dL. He was taken for an EGD on the second day of admission, which demonstrated a large ulcer (20 mm in diameter) in the distal esophagus, along with grade C esophagitis, a medium-sized hiatal hernia, and localized moderate inflammation in the gastric antrum (Figure 4). Moreover, it was noted that no signs of bleeding were present, but he did have a healing scar in the greater curvature of the stomach from a previous gastrostomy tube (5 mm) that was removed two months prior and resulted in healthy scar tissue (Figure 5). A CT of the abdomen on the same day as the EGD showed a large pneumoperitoneum and diverticulosis (Figure 6). However, no perforation or gas leak was noted on a subsequent upper GI series with gastrografin (Figures 7-10). Although a large pneumoperitoneum was found, the patient did not endorse any symptoms or complaints. A repeat CT of the abdomen several days later showed an unchanged pneumoperitoneum and a fluid collection near the healing scar of the previous PEG site that was 7.6 cm × 5 cm and contained debris and air (Figure 11). This was thought to be the cause of the pneumoperitoneum noted earlier. Interventional radiology was consulted, but they opted not to drain the fluid then. A third CT of the abdomen one week later showed decreased fluid collection and an improvement in the size of the pneumoperitoneum (Figure 12). The surgical team elected to continue monitoring for any changes and decided to take a more conservative approach to managing this patient due to the changes noted on serial CT scans. After several months, repeat imaging showed a resolution of the pneumoperitoneum and a near-complete resolution of the fluid collection (Figure 13).

## Discussion

Pneumoperitoneum is a common finding in patients who undergo percutaneous endoscopic gastrostomy due to the high pressure required to insert the gastric tube while simultaneously applying endoscopic air insufflation [1]. Most pneumoperitoneums are not threatening and do not present with any significant findings, and they tend to resolve spontaneously [1]. When characterizing pneumoperitoneum, it is most commonly referred to as either surgical or nonsurgical [2]. Non-surgical pneumoperitoneum is also called spontaneous or misleading pneumoperitoneum. Our patient presented with a nonsurgical pneumoperitoneum, which failed to present with any positive findings regardless of size, even in cases with mild abdominal discomfort [3]. These positive signs include fever, leukocytosis, or peritoneal signs [3].

Understanding that nonsurgical pneumoperitoneum has a variety of causes can aid in the clinical discussion among specialties and management. Specifically in the abdominal cavity, spontaneous pneumoperitoneum can be caused by open abdominal surgery, laparoscopic cholecystectomy, spontaneous bacterial peritonitis, or blunt abdominal trauma [3]. Other less commonly reported causes include barium enema with rectal insufflation, splenic embolization procedures, and gastric emphysema [3]. As mentioned previously, the insertion of a gastric tube in a PEG procedure has been shown to lead to abdominal pneumoperitoneum and has been reported to occur in 30-40% of cases [2]. Still, to the best of our knowledge, this is the only published case report reporting the removal of a gastric tube that led to abdominal pneumoperitoneum. We theorize that the fluid and air buildup near our patient’s gastric scar led to the abdominal pneumoperitoneum. Management of this nonsurgical or spontaneous pneumoperitoneum includes carefully monitoring the size of the pneumoperitoneum and the patient's presenting symptoms. Especially when the patient fails to present with peritonitis and presents without any distress, conservative management should be considered [4]. If a patient progresses to have a fever, leukocytosis, and peritoneal signs, then by definition, the pneumoperitoneum is no longer categorized as nonsurgical, and prompt surgical intervention may be warranted.

In our patient, we believe it is essential to also focus on the fact that he had colonic strictures before the PEG procedure and after removing his gastrostomy tube. No other documentation has correlated colonic strictures and PEG procedures with pneumoperitoneum. We would like to raise the question of the role of screening patients for colonic strictures before inserting or removing a gastric tube. Patients with a history of colonic strictures might have undergone colonic dilation or multiple colonoscopies, all of which could have led to the development of a pneumoperitoneum. No pneumoperitoneum was ever found in our patient prior to the gastrostomy tube removal, and no colonic perforation was noted afterward. Further research is warranted to investigate the possibility of the development of pneumoperitoneum after gastric tube removal in patients with a documented history of colonic strictures.

## Conclusions

Pneumoperitoneum, or free air within the peritoneal cavity, is an understood and well-documented complication of PEG procedures. All pneumoperitoneums are not alike, and the location and presentation of a pneumoperitoneum dictate its management. In our case, the patient failed to present with any peritoneal signs or signs of infection and was labeled to have a nonsurgical pneumoperitoneum, for which conservative management was warranted. Patients who fail to present with leukocytosis or peritoneal signs often do not need surgical correction, as the pneumoperitoneum resolves spontaneously. Our patient was never noted to have a pneumoperitoneum after inserting his gastrostomy tube, but rather, it formed after removing the gastrostomy tube, which warranted this case to be shared. The decision of how to treat our patient was based on guidelines for pneumoperitoneums caused by other etiologies, and our patient's pneumoperitoneum resolved over the course of time without any intervention.
